# Extracellular calcium regulates keratinocyte proliferation and HPV 16 E6 RNA expression *in vitro*

**DOI:** 10.1111/apm.12227

**Published:** 2014-02-12

**Authors:** Aaro Turunen, Stina Syrjänen

**Affiliations:** 1 Department of Oral Pathology, Institute of Dentistry, University of TurkuTurku, Finland; 2 Department of Pathology, Turku University HospitalTurku, Finland

**Keywords:** HPV, E6, E2, calcium, differentiation

## Abstract

Human papillomaviruses (HPV) are known to immortalize oral keratinocytes *in vitro,* but the underlying mechanisms causing the following resistance to differentiation remain unclear. We investigated the effect of extracellular calcium on the proliferation of HPV16-positive keratinocytes and on the mRNA expression of the viral E6-oncogene. HPV16-positive hypopharyngeal carcinoma cells (UD-SCC-2), spontaneously immortalized- (HMK) and HPV16 E6/E7-immortalized human gingival keratinocytes (IHGK) were grown for 3, 6 and 9 days in Keratinocyte Serum-free Medium with calcium concentrations ranging from 0 mM to 6 mM. Calcium concentrations up to 0.09 mM increased cellular proliferation, which decreased at higher concentrations. A shift of calcium concentration from 0 to 4 mM increased E6 expression in UD-SCC-2 cells 2.4-fold by day 9. Simultaneously, E2 expression increased. The most significant upregulation of E6 and E2 expressions was observed at day 9, grown in high-calcium media and the increase in E6 expression coincided with an increase in involucrin expression, likely indicating cell differentiation. Despite this, HPV-positive cells continued to proliferate even at high-calcium media in contrast to HPV-negative cells. Overexpression of E6 mRNA may be an important feature of HPV16-positive cells to resist the natural calcium gradient in differentiating keratinocytes allowing cell proliferation.

Human papillomavirus (HPV) type 16 is the most potent carcinogenic virus at several locations of the body especially at the anogenital tract, oral cavity and pharynx. In addition, there are 11 other high-risk HPVs, which have sufficient evidence for causality of cervical cancer 1–4. High-risk HPV types cause malignant progression mainly via their oncogenes E6 and E7 5. Normally, HPV maintains its genome episomally in the nucleus and the expression of E2 protein modulates the levels of E6 expression 6. In carcinomas, HPV is often integrated with loss of viral episomes. Viral integration can lead to disruption of E2 gene, overexpression of E6 mRNAs and cell cycle dysregulation 7. The E6 oncoprotein interferes with cell cycling by degrading cellular p53, the so-called guardian of the genome, resulting in resistance to terminal differentiation and cell death. The biological effects of E6 are mediated by E6-associated protein (E6-AP) along with E6-binding protein (E6-BP), which is a cellular calcium-binding protein 8–10.

Differentiation-specific cellular factors link the HPV gene expression to host keratinocyte differentiation 4,11 not present in conventional monolayer cultures and hinder the completion of the HPV life cycle. Extracellular calcium is an important factor involved in terminal differentiation 12 and is used to increase differentiation in cell cultures. *In vitro* experiments demonstrate that increases in extracellular calcium of the growth medium also increase intracellular calcium concentrations 13. *In vivo*, basal keratinocytes are exposed to low-calcium concentration, while a progressively increasing calcium gradient is present in the upper layers of epithelium until the superficial stratum corneum at which point calcium rapidly declines 14,15. Mucosal or skin keratinocytes are often maintained in a basal, proliferative state by culturing in growth media containing lower than 0.1 mM calcium. Differentiation can then be induced by elevating the calcium concentration to over 1 mM of extracellular calcium. This ‘calcium switch’ causes the cells to exit from cell cycle and commits them to terminal differentiation. This method has been widely used to study keratinocyte differentiation 16,17 and is used to select for cells transformed with HPV16 18. It is likely that HPV-positive cells are able to resist calcium-induced terminal differentiation signals and E6 proteins seem to be central mediators of these effects 19. Downregulation of HPV16 E6 oncogene mRNA expression has also been reported earlier to be associated with keratinocyte differentiation induced by added 1.0 mM calcium 20.

Here, we assessed the effects of extracellular calcium on keratinocyte cell growth and HPV16 E6 oncogene expression levels of HPV-positive immortalized cell lines and compared the results with those of a HPV-negative immortal cell line. We were especially interested in studying cell lines derived from oral mucosa as there are insufficient data on HPV16 in *in vitro* differentiation-inducing environment. Increasing amount of clinical research has recently focused on HPV as a cause of oral and oropharyngeal cancer, but *in vitro* studies on oral keratinocytes are scanty, although it is known that HPV16 is also the most common high-risk HPV type in asymptomatic oral infections. Because the HPV oncogene E6 is expressed in undifferentiated basal- or parabasal cells where the calcium concentrations are low, we hypothesized that HPV16-positive cell lines would express the HPV16 E6 oncogene when grown in a medium with a low-calcium concentration. On the contrary, HPV16 E6 mRNA expression levels are expected to lower with increasing calcium concentrations. Increased calcium concentration should also restrict the proliferation of oral keratinocytes at concentrations over 1 mM because of the growth/differentiation switch. We report that HPV16 E6/E7-immortalized keratinocytes responded poorly to the calcium switch and continued to proliferate even in high-calcium medium, contradictory to HPV-negative cells. This response might be related to increase in HPV16 E6 and E2 mRNA expression. This information would be vital for understanding the specific mechanisms of resistance to differentiation and later progression towards carcinogenesis in the HPV-positive oral keratinocyte.

## Methods

### Cell lines

Three cell lines, UD-SCC-2, IHGK, HMK, were used in this study. UD-SCC-2 is a HPV16 DNA-positive cancer cell line derived from a hypopharyngeal carcinoma 21. In this cell line, there are approximately 600 copies of HPV16 per cell. IHGK cell line represents human gingival epithelial cells immortalized by HPV16 E6 and E7 oncogenes 22. HMK cell line is a spontaneously immortalized gingival keratinocyte cell line 23 that does not contain any HPV DNA.

### Cell cultures

First, the cells were thawed from liquid nitrogen and passaged in the medium they were normally cultured in. UD-SCC-2 cells were grown in DMEM (Gibco, Grand Island, NY, USA) supplemented with 1% non-essential amino acids, 2 mM L-glutamine, 100 g/mL streptomycin, 100 IU/mL penicillin and 10% foetal calf serum. IHGK and HMK cells were grown in Keratinocyte Serum-free Medium (KSFM by Gibco) with 0.045 mM and 0.09 mM calcium chloride, respectively, supplemented with human recombinant EGF (0.1–0.2 ng/mL) and bovine pituitary extract (20–30 μg/mL). The cells were grown in 80 cm^2^ Nunclon flasks (Sigma-Aldrich, St. Louis, MO, USA) for three passages and were then trypsinized, and plated onto 6-well plates at a concentration of 50 000 cells/well in regular KSFM (with 0.09 mM calcium). The experiment was done in triplicates and repeated twice.

### Exposure to calcium

After initial plating, the cells were allowed to adhere for 24 h before the calcium exposure. The cell lines were exposed to different calcium concentrations by adding calcium chloride to calcium-free KSFM to achieve the following calcium concentrations of 0 mM, 0.045 mM, 0.09 mM, 1.8 mM, 3 mM and 4 mM. In addition, the UD-SCC-2 cell line was also exposed to 5 mM and 6 mM calcium in KSFM, as these cells tolerated higher calcium concentrations than the immortalized cells. The exposed cell cultures were harvested at day 3, 6 and 9 by trypsinizing the cells and then the cells were counted in Bürker cell chambers for the growth curves. The medium was changed every 48 h during the cultures. After trypsinization, the cells were suspended in TRIzol reagent (Invitrogen, Paisley, UK) for RNA extraction and real-time PCR analysis.

### Real-time PCR

mRNA from the harvested cells was extracted using the TRIzol reagent according to the manufacturer’s protocol. First-strand cDNA was synthesized using First-strand cDNA Synthesis Kit (GE Healthcare, Little Chalfont, UK) using pd (N)6 random hexamers for priming. Reactions contained 1 μg of total RNA in volume of 15 μL. The RT reactions were performed in reaction volume of 25 μL containing 15 ng sample with Universal MasterMix and custom TaqMan® Gene Expression assays (Applied Biosystems, Foster City, CA, USA): HPV16 E6 (E6 probe (6-FAM)-CAGGAGCGACCCAGAAAGTTACCACAGTT-(MGBNFQ), E6 forward primer GAGAACTGCAATGTTTCAGGACC, E6 reverse primer TGTATAGTTGTTTGCAGCTCTGTGC), HPV16 E2 (E2 probe (VIC)-CACCCCGCCGCGACCCATA-(MGBNFQ), E2 Forward primer AACGAAGTATCCTCTCCTGAAATTATTAG, E2 reverse primer CCAAGGCGACGGCTTTG) 24, Involucrin (TaqMan Gene Expression Assays Hs00846307_s1, Applied Biosystems, Foster City, CA, USA) or GAPDH (Applied Biosystems) using the Taqman 7900HT Fast Real-Time PCR System (Applied Biosystems) according to the manufacturer’s protocol. The reaction conditions were 2 min at 50 °C, 10 min at 95 °C, and a two-step cycle of 95 °C for 15 s and 60 °C for 60 s for a total of 40 cycles.

Standard curves were prepared by amplification of a 5-fold dilution series of 200 ng to 6.25 ng of cDNA from SiHa cells and cDNAs extracted from two different HPV16-positive cervical carcinoma samples. For Involucrin, the standard curves were prepared by amplification of a 5-fold dilution series of 200 ng to 6.25 ng of cDNA derived from condyloma samples. All reactions were performed as triplicates. The results were normalized against GAPDH mRNA levels.

### Statistical analyses

We used SPSS 19 version 19.0.0.1 (IBM Corp. Released 2010. IBM SPSS Statistics for Windows, Version 19.0. Armonk, NY, USA). The statistical analysis was performed by first applying the univariate general linear model (GLM) on the triplicate normalized values from the two repeat experiments. The specific adjusted R-squared values and significances of calcium concentration on the observed gene expressions are given in results section. The mean difference was considered significant when p < 0.05. For group-to-group comparisons, Levene′s test for equality of variances was employed. Subsequent t-testing for equality of means, adjusted for equal- or unequal variances between specific samples as determined by the Levene′s test, was used to determine significant differences between gene expressions.

## Results

### Cell proliferation

**Figure 1:**
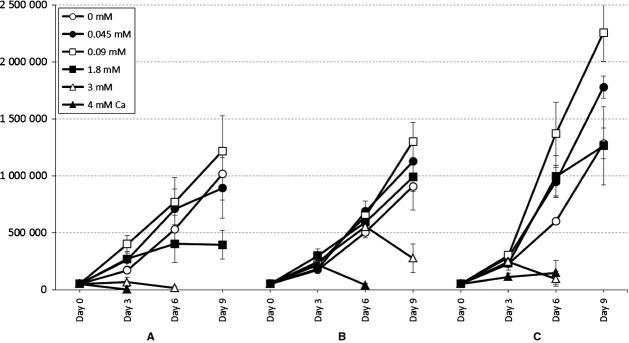
The growth curves of cell lines grown at different calcium concentrations are shown. The calcium concentrations are presented as millimoles per litre (mM). Fig1A HMK cell line. Fig1B UD-SCC-2 cell line. Fig1C IHGK cell line.

Figure1 summarizes the growth curves of tested cell lines grown in media with increasing calcium concentrations from 0 mM to 4 mM. Exposure with increasing extracellular calcium concentration resulted in increasing cell proliferation until the concentration of 0.09 mM in all cell lines studied as shown in Fig.1. At calcium concentrations above the previously mentioned ‘calcium switch’ (>0.1 mM), the cell proliferation generally declined. However, the HPV E6/E7-immortalized keratinocytes responded poorly to the calcium switch and continued to proliferate even in high-calcium medium up to 3 mM, contradictory to HPV-negative HMK cells. The number of HMK cells in high-calcium medium declined after 6 days, probably due to the differentiation and shedding to the medium. HMK cells rapidly died in 4 mM calcium. On average, only 1000 cells were remaining per well after 3 days of culture. Obvious differences between cell lines were also found at 1.8 mM calcium, where HMK cells′ proliferation clearly slowed by day 9 when compared with that at lower calcium concentrations. Proliferation of UD-SCC-2 and IHGK at 1.8 mM calcium remained almost unchanged when compared with that found at 0 mM calcium and the growth curve of UD-SCC-2 cells rose fastest at 1.8 mM between days 6 and 9 (Fig.1). UD-SCC-2 cells tolerated Ca^2+^ concentrations of 3 mM and 4 mM better than the immortal cell lines, but did not proliferate at 5 mM and 6 mM calcium concentrations (data not shown). However, accurate counting of the cells in calcium concentrations of 3 mM or higher was not possible because the cells could not be trypsinized free from the wells due to their strong adhesion after 3 days of culture for HMK, 6 days for IHGK and 9 days for UD-SCC-2. Harvesting at these time points was achievable only by gently scraping with a sterile cell scraper. Also, a precipitation was noted in medium from day 3 onwards for both immortalized cell lines and from day 6 onwards for UD-SCC-2 when calcium was added at final concentrations of over 3 mM.

### Analysis of HPV E6 and E2 mRNA levels

**Figure 2:**
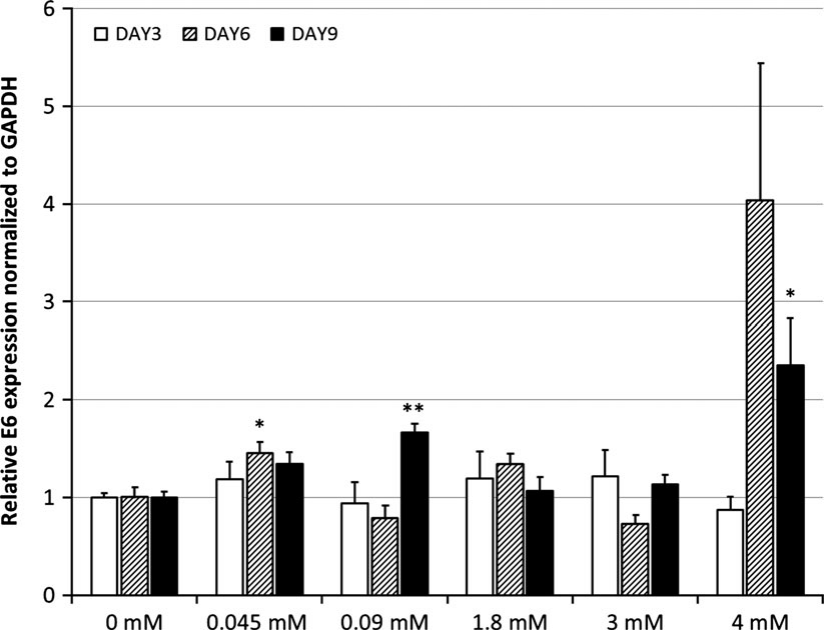
UD-SCC-2 E6 mRNA expression. The mean expression levels of HPV 16 E6 mRNA of UD-SCC-2 cells are calculated from triplicate repeated analyses. The results are related to the GAPDH-normalized E6 mRNA values without any calcium (Student′s t-test with unequal or equal variances, as determined by the Levene′s test beforehand). The p-values are shown as p < 0.05 = * and p < 0.01 = **. The significance for day 6 samples in 4 mM was p = 0.083 and for day 9 in 0.045 mM p = 0.065.

The expression of E6 mRNA in UD-SCC-2 cells grown in media at different calcium concentrations is depicted in Fig.2. Increases in the calcium concentration of the medium resulted in an elevated expression of the HPV16 E6 mRNA. A significant increase was found in cell cultures grown in media with 0.045 mM calcium at day 6 (p = 0.044) and with 0.09 mM and 4 mM calcium at day 9 (p = 0.004 and p = 0.018 respectively). Mean E6 mRNA levels were highest at day 6 in 4 mM, but this upregulation was not significant (p = 0.083).

**Figure 3:**
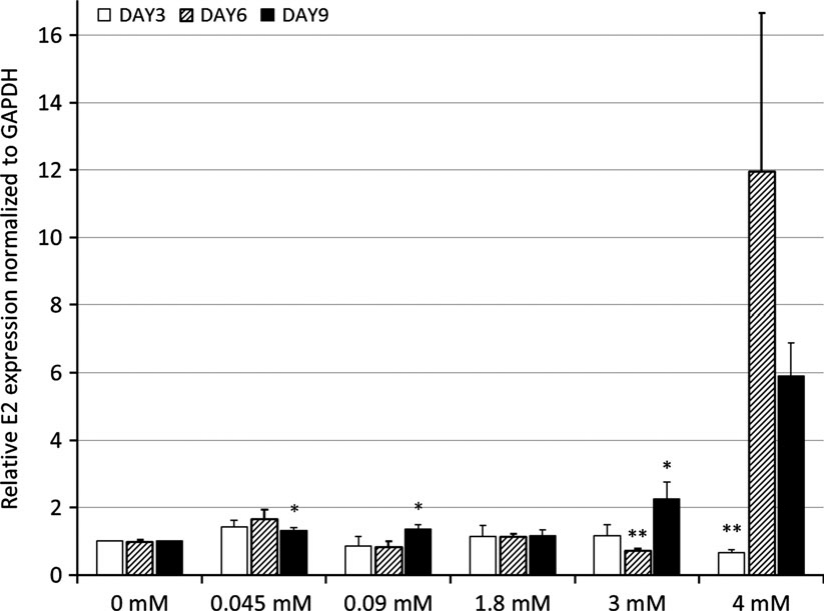
The levels of E2 mRNA expressed in UD-SCC-2 cells calculated similarly as in Fig.2 are shown. The significance levels for day 6 samples in 0.045 mM is p = 0.055. For day 6 and 9 samples in 4 mM p-values are 0.075 and 0.059 respectively. The p-values are shown as p < 0.05 = * and p < 0.01 = **.

The effect of calcium concentration on HPV16 E2 expression in UD-SCC-2 cells is shown in Fig.3. A significant upregulation was found at day 9 with calcium concentration of 0.045 mM, 0.09 mM and 3 mM (p = 0.03, p = 0.042, p = 0.07 respectively). At higher calcium concentrations 3 mM and 4 mM, HPV16 E2 mRNA levels first declined as evident in Fig.3 (p = 0.01 and p = 0.008 respectively).

**Figure 4:**
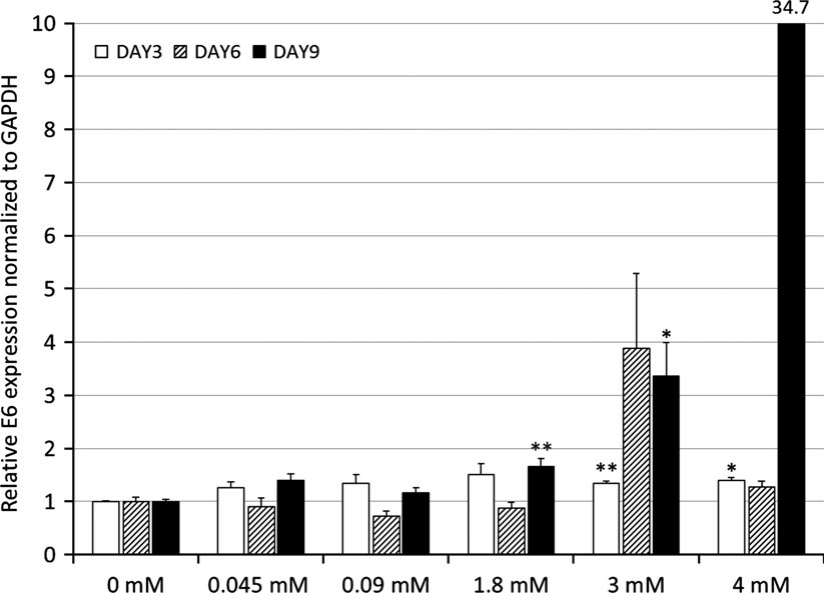
The levels of HPV 16 E6 mRNA expressed by IHGK cells as related to the exposure to different calcium concentrations are shown. Graphs are presented and calculated similarly as in Fig.2. At day 9 in 4 mM calcium, the average is off the chart and shown as a number above the bar. The p-value for this point is 0.143. The p-values are shown as p < 0.05 = * and p < 0.01 = **.

E6 mRNA expression affected by calcium in HPV 16 E6/E7 immortalized gingival keratinocytes (IHGK) is shown in Fig.4. The highest statistically significant upregulation was found when comparing cultures without calcium with those with 3 mM calcium (p = 0.02) at day 9. The mean IHGK E6 mRNA levels were highest at day 9 in the cells grown with 4 mM calcium, but this difference was statistically non-significant due to the wide variation in mRNA values in individual wells (p = 0.143).

We also used univariate general linear modelling, which revealed that calcium concentration of the medium was a significant regulating factor for E6 expression in both UD-SCC-2 and IHGK cell lines and for E2 expression in UD-SCC-2 cells. For UD-SCC-2, the test of between-subjects effects revealed extracellular calcium as significant factor for E6 expression with 5 degrees of freedom (df), F = 29.243, p < 0.0001 and adjusted R squared = 0.893. For UD-SCC-2 E2, the values were 5 df, F = 71.427, p < 0.0001 and adjusted R squared = 0.946. For IHGK E6 expression, the values were as follows: 5 df, F = 3.507, p = 0.007 and adjusted R squared = 0.565.

### Analysis of involucrin mRNA expression

**Figure 5:**
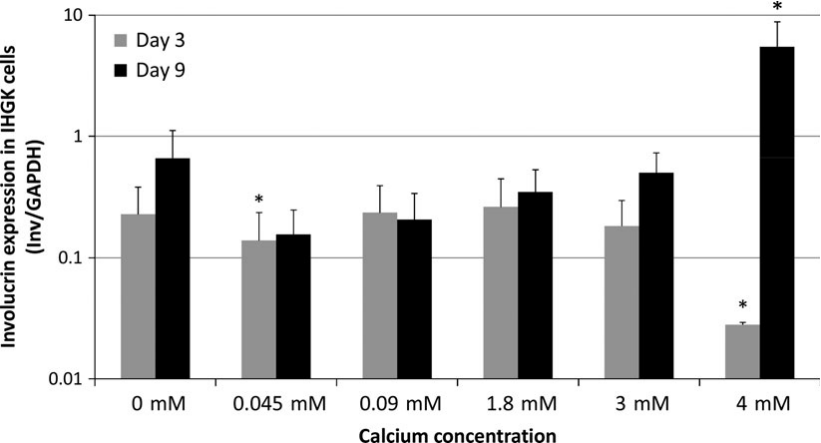
Involucrin mRNA expression in IHGK cells as related to the exposure to different calcium concentrations. The graphs are calculated similarly as in Fig.2. The p-values are shown as * = p < 0.05.

**Figure 6:**
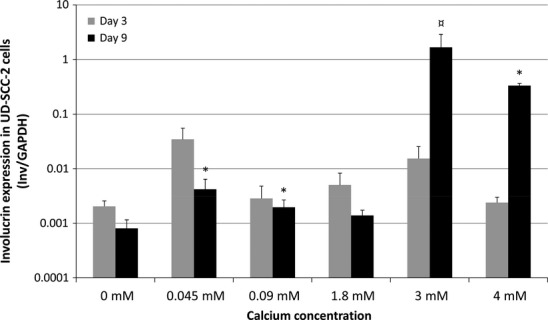
Involucrin mRNA expression in UD-SCC-2 cells as related to the exposure to different calcium concentrations. Graphs are presented and calculated similarly as in Fig.5. At day 9 at 3 mM calcium, the mark ¤ represents the p-value of 0.055 of borderline significance. The p-values are shown as p < 0.05 = *.

Figures5 and 6 summarize the mRNA expression of involucrin in IHGK and UD-SCC-2 cells, respectively, at 3 and 9 days of culture. Expression of involucrin in HPV16-transformed IHGK cells declined or remained at the same level at day 3 after calcium exposure at different concentrations except in the cells grown at 0.045 mM and 4 mM calcium concentrations in which the expression downregulated (p = 0.0001 and p = 0.003 respectively). At day 9, the expression of involucrin was higher than that found at day 3 except in the cells grown in a calcium-free medium. There was a statistically significant upregulation of involucrin expression at 4 mM calcium when compared with that at 0 mM (p = 0.025).

In UD-SCC-2 cells, no significant differences in involucrin expression were found at day 3. At day 9, increasing the calcium concentration from 0 - to 0.045- or 0.09 mM increased the involucrin expression (p = 0.02 and p = 0.0001 respectively). When the calcium concentration was further increased, there was a 2064- and 401-fold upregulation of involucrin expression at 3 mM and 4 mM concentrations (p = 0.055 and p = 0.0001) respectively.

Calcium concentration of the medium was a significant regulator of involucrin expression in IHGK and UD-SCC-2 cells also when assessed by univariate general linear modelling. For IHGK, the test of between-subjects effects revealed extracellular calcium as significant factor for E6 expression at days 3 and 9 with 5 degrees of freedom (df), F = 41.46 and 211.97, p < 0.0001 for both and adjusted R squared = 0.91 and 99 respectively. For UD-SCC-2 cells, these values for days 3 and 9 were 5 df, F = 226.96 and 40.34, p < 0.0001 for both and adjusted R squared = 0.987 and 0.933 respectively.

## Discussion

This study showed that the HPV-positive cell lines were more resistant to calcium-induced cessation of cell proliferation than the HPV-negative immortal cell line. One can speculate that cellular differentiation signal due to the increased calcium concentration is likely responsible for the persistent upregulation in E6 mRNA transcription we observed in our cultures. This would lead E6 to interfere with the cessation of cell proliferation as is evident from the growth curves (Fig.1) where steady cell proliferation is observed from day 0 onward to day 9, also irrespective of confluence. Already in 1989, Yasumoto and coworkers showed 20 that HPV can induce cell proliferation and resistance to differentiation. This is also supported by the data from Wilding and coworkers 18, as they selected successfully HPV16 E6/E7-transfected keratinocytes by increasing the calcium content of the medium. Here, we provided more evidence that the evasion of differentiation is related to E6 transcription as has been demonstrated earlier in keratinocytes of the foreskin, where HPV16 E7 had no direct specific effect on involucrin expression 25.

Micallef and coworkers 26 reported that HaCat cells had no detectable expression of keratins 1 or 10 mRNAs at day 1, but increasing expression levels were found at day 3 and 6 after exposure to calcium concentration of 1.2 mM. These results combined with ours indicate that changes in calcium-induced gene expression are also time dependent, manifesting only after days in culture. Micallef and coworkers also reported the finding that HaCat cells resisted calcium-induced cessation of proliferation. This was hypothesized to be caused by a mutation affecting the p21, PKC or the calcium receptor (CaR) pathways. As PKC is a key factor in HPV life cycle progression 27 and is a central mediator in calcium signalling pathways, it is tempting to think that altered HPV16 E6 mRNA expression found in our study disrupts these same pathways leading to very similar effects, for example the E6 protein interfering with p53, which would in turn downregulate p21 leading to continuing proliferation. To confirm that the changes we observed in HPV gene expressions were caused by a differentiation-inducing environment as a result of increasing calcium concentrations, we were able to show that the increase in the expression of involucrin gene, a marker of differentiation, was similarly related to increase in calcium concentration in IHGK and UD-SCC-2 cells. This also needed time to develop and also only manifested at the day 9 time point.

Yasumoto and coworkers 20 demonstrated that keratinocyte differentiation induced by 1 mM calcium exposure was associated with down-regulation of HPV16 E6/E7 transcription in HPV E6/E7 immortalized primary skin keratinocytes. This decrease was attributed to decrease in p97-promoter expression. However, the oncogene expression may be maintained, although at a lower level, by p441 and p542-promoter expression in cases in which p97 downregulation is induced by added high levels of calcium as stated by Hansen et al. 28. Contradictory to these reports, our results showed that the calcium switch eventually lead to increased E6 transcription as the difference in the mRNA levels was not apparent before the day 9 in a high-calcium medium (Fig.4). Hansen and coworkers 28 analysed their samples at day 5, not necessarily allowing enough time for the late effects of calcium to completely manifest. This is also supported by the work of Deyrieux and Wilson 29 who reported that calcium-induced differentiation occurred in 144 h after switching the calcium concentration. It should be mentioned that the cell lines in question are also very different, harbouring different states of the HPV16 genome. Hansen et al. used the cervical cancer cell line SiHa having 1-2 integrated HPV16 genomes, whereas we used the hypopharyngeal cancer cell line UD-SCC-2 that contains approximately 600 copies of HPV16 and the non-transformed IHGK cell line that has only been transfected with E6/E7 oncogenes.

IHGK and UD-SCC-2 cells survived the high exposure of calcium (3 mM and 4 mM) as indicated by continued E6 mRNA transcription and observations under the light microscope (data not shown). Only concentrations of 5 mM and higher quickly resulted in UD-SCC-2 cell death as was evident also by cell morphology. As most mammalian cells can tolerate an osmolality of 260 to 350 mOsm/kg 30, the cell exposure to 1.8 mM–6 mM of CaCl should not have caused direct osmolality induced cell death in this experiment. However, as precipitations in the media occurred when calcium concentration was higher than 3 mM, we cannot estimate the accurate final calcium concentration in the media. Interestingly, upregulation of E2 and E6 often coincided with this precipitation. This phenomenon needs to be studied more closely in the future experiments.

One possible explanation of the UD-SCC-2 cell death after high-calcium exposure was the coinciding increase in HPV16 E2 mRNA expression. E2 protein is linked with cell senescence when expressed in HPV 18-positive HeLa cancer cells and HaCat cells 31,32. Accordingly, the high E2 expression in UD-SCC-2 could surpass the effect of E6 and E7, leading to the observed senescence and apoptosis. IHGK cells are transformed only with HPV16 E6/E7 and do not have the regulatory functions of E2 gene. This may be one reason to the high proliferation rate of IHGK cells after calcium exposure exceeding even the proliferation rates of UD-SCC-2 cancer cells as observed in Fig.1. Also, as UD-SCC-2 cells are cancer cells, their capacity to differentiate is altered. UD-SCC-2 cells presented with elevated E6 expression already after low increases in calcium concentration. Despite this, E6 expression was similarly affected by calcium at differentiation-inducing levels, as it was in IHGK cells in which E2 is not present. IHGK cells may have higher proliferation due to their lack of regulating E2, but these cells are fast growing under normal culture conditions and therefore no firm conclusions can be drawn regarding the lack of E2 in this experimental setting. It is possible that HPV-positive cancer cells may still retain some of their capacity to respond to differentiation-inducing calcium signals. This is supported by the results of Hansen and coworkers 28 who reported calcium-induced differentiation-related gene expression in a HPV-positive cervical cancer cell line and is further supported by our finding that involucrin mRNA expression is elevated at high calcium in UD-SCC-2 cells; even though as carcinoma cells, their capability for differentiation should be impaired.

To conclude, we have demonstrated that HPV-immortalized cells can resist the effect of high-calcium concentration *in vitro* possibly via E6 transcription. E6 mRNA expression might be an important feature of HPV16-positive cells to resist the natural calcium gradient in differentiating keratinocytes allowing their proliferation and possibly later selection towards malignancy. However, as calcium is a core element in several molecular pathways, more research is needed to elucidate the calcium-activated pathways in HPV-induced transformation.
